# New-Onset Atrial Fibrillation in the Setting of COVID-19 Infection Is a Predictor of Mortality in Hospitalized Patients: CovAF-Study

**DOI:** 10.3390/jcm12103500

**Published:** 2023-05-16

**Authors:** Mariana S. Parahuleva, Lukas Harbaum, Nikolaos Patsalis, Nikoleta Parahuleva, Christian Arndt, Ulrich Lüsebrink, Bernhard Schieffer, Julian Kreutz

**Affiliations:** 1 Department of Cardiology, Angiology and Intensive Care Medicine, Philipps University of Marburg, 35043 Marburg, Germany; 2 Department of Obstetrics and Gynecology, Medical University of Plovdiv, 4002 Plovdiv, Bulgaria; n_nikoleta1986@abv.bg; 3 Department of Anesthesia and Intensive Care Medicine, Philipps University of Marburg, 35043 Marburg, Germany; christian.arndt@uk-gm.de

**Keywords:** COVID-19, atrial fibrillation, mortality

## Abstract

Recent studies show that hospitalized COVID-19 patients have an increased incidence of arrhythmia, especially atrial fibrillation (AF). This single-center study included 383 hospitalized patients with positive polymerase chain reaction tests for COVID-19 from March 2020 to April 2021. Patient characteristics were documented, and data were analyzed for episodes of AF on admission or during the hospital stay, intrahospital mortality, need for intensive care and/or invasive ventilation, inflammatory parameters (hs-CRP, IL-6, and procalcitonin), and differential blood count. We demonstrated that in the setting of hospitalized cases of COVID-19 infection, there is an incidence of 9.8% (*n* = 36) for the occurrence of new-onset AF. Furthermore, it was shown that a total of 21% (*n* = 77) had a history of episodes of paroxysmal/persistent AF. However, only about one-third of patients with pre-existing AF had relevant documented tachycardic episodes during the hospital stay. Patients with new-onset AF had a significantly increased intrahospital mortality compared to the control and the pre-existing AF without rapid ventricular rate (RVR) group. Patients with new-onset AF required intensive care and invasive ventilation more frequently. Further analysis examined patients with episodes of RVR and demonstrated that they had significantly elevated CRP (*p* < 0.05) and PCT (*p* < 0.05) levels on the day of hospital admission compared to patients without RVR.

## 1. Introduction

The coronavirus disease 2019 (COVID-19) pandemic is caused by Severe Acute Respiratory Syndrome Coronavirus type 2 (SARS-CoV-2), a beta coronavirus. It is a highly infectious virus that spreads mainly by respiratory droplet infection. It can also be transmitted through contact with contaminated surfaces and objects. Symptoms of COVID-19 may include fever, cough, shortness of breath, fatigue, body aches, loss of taste or smell, and sore throat. In severe cases, the virus can cause pneumonia, which can develop into life-threatening acute respiratory distress syndrome (ARDS) and require invasive ventilation [1]. It is well known that the clinical manifestations of the disease caused by COVID-19 vary widely, ranging from asymptomatic infection to severe respiratory failure with multiorgan failure and death [2]. To date, there is no specific antiviral treatment for COVID-19. 

SARS-CoV-2 can enter cells via a spike protein. It is located on the surface of the virus and binds to angiotensin-converting enzyme 2 (ACE2), which is found on the surface of human cells, particularly in the lungs, heart, and kidneys. It is composed of two subunits: S1, which contains the receptor-binding domain that binds to ACE2, and S2, which helps the virus fuse with the host cell membrane and enter the cell. Small genetic changes in the spike proteins can lead to increased pathogenicity of the virus and the formation of different virus variants [3,4]. During the pandemic so far, there have already been several variants with different transmissibility and varying severity of illness. A high mortality rate was observed worldwide, especially in the first months of the pandemic. The availability of vaccines as of December 2020 had a positive impact on the COVID-19 pandemic, as a significant decrease in symptomatic infections, severe courses, and SARS-CoV-2-related deaths was observed [5]. In addition to protection by vaccination, meta-analyses have shown that naturally acquired antibodies against SARS-CoV-2 can significantly reduce the risk of subsequent infection and that the severity of the disease is usually milder upon re-infection. However, it is still unclear to what extent this protection by naturally acquired antibodies persists [6].

It is now known that various organ systems are affected in addition to pulmonary manifestations, and SARS-CoV-2 may have relevant effects on the cardiovascular system. Here, damage and inflammation of the heart muscle may occur, leading to cardiac arrhythmias, heart failure, or even sudden cardiac arrest. Acute myocardial damage, as inferred from elevated cardiac troponin levels, has been reported in 8% to 62% of patients hospitalized with COVID-19 in various studies [7]. In addition, COVID-19 has been associated with an increased risk of blood clots that can lead to myocardial infarction, stroke, or other cardiovascular events. New waves of COVID-19 continue to emerge, but meanwhile, we better know how to prevent severe courses in patients with multiple comorbidities and how to adjust therapeutic care to improve future outcomes [8]. 

Patients with pre-existing underlying cardiovascular diseases and corresponding comorbidities such as diabetes, obesity, and hypertension showed a more severe course of the disease and, subsequently, an increased mortality rate during the current SARS-CoV-2 pandemic. In one of the largest global surveys available, which included 4526 hospitalized patients with COVID-19 around the world, 18.27% of patients developed arrhythmias due to COVID-19 [9]. Atrial fibrillation (AF), especially, is one of the most important conditions contributing to an increased risk of severe disease [10,11,12]. AF can be triggered by inflammatory processes in the setting of sepsis or pneumonia, as inflammation in the body can cause changes in the electrical properties of the heart that can lead to the development of AF [13]. The new evidence showed that AF is independently associated with higher mortality. Moreover, a recent meta-analysis showed that patients admitted for COVID-19 and comorbid AF had significantly higher all-cause mortality as compared to those without AF [14]. Furthermore, the patients hospitalized with COVID-19 and pre-existing AF had a higher risk of developing acute respiratory distress syndrome during hospitalization without any difference in the risk of death [15]. It has been suggested that the severe acute respiratory syndrome COVID-19 may directly provide the pathogenesis of AF through atrial inflammation as well as an alteration in the atrial electrophysiology and through dysregulation in cellular angiotensin-converting enzyme 2 receptors [16]. Interestingly, there are some other hypotheses that have been postulated to explain the pathophysiology of arrhythmia during COVID-19. These include the role of sialic acid, CD147, IL-18, and the spike protein [17]. In addition, pneumonia can cause fluid buildup in the lungs, which can strain the heart and increase the risk of developing AF. Furthermore, it could be parallelism with community-acquired pneumonia (CAP) patients, as reported in other meta-analyses [18]. The key mechanisms and potential predictors of the incidence of AF are inflammatory responses in the atrial myocardium as well as adipose-tissue inflammation [19,20]. On the other hand, new-onset AF in patients with COVID-19 may be a factor that predicates other adverse cardiovascular diseases rather than an independent factor of mortality [21]. Moreover, despite higher anticoagulation use in patients with COVID-19 and new-onset AF, ischemic stroke and embolic events were more common, possibly due to the presence of additional risk factors such as hypertension and peripheral vascular disease [22]. 

Despite the research findings to date and the advancing treatment options for COVID-19, it is important to better understand the risk factors for severe disease progression. This is particularly true for vulnerable patient groups such as those with cardiovascular disease. In this regard, few studies to date have directly compared the effects of COVID-19 on patients with different types of cardiovascular disease [23]. Understanding outcomes associated with COVID-19 in hospitalized patients with cardiovascular disease is important for establishing guidelines for infection control, resource planning, and clinical care. Understanding the epidemiology of COVID-19 and emerging cardiovascular diseases, particularly AF, is also relevant to the development and evaluation of new therapeutic consequences in cardiovascular medicine and electrophysiology. In this study, we aimed to determine the extent to which the occurrence of AF (new-onset/paroxysmal/persistent) influences disease progression in the setting of COVID-19. To analyze patients among new-onset AF, pre-existing AF, and control groups with a documented history of sinus rhythm, individual patient characteristics are described. 

## 2. Materials and Methods

### 2.1. Study Design, Case Definition, and Control Selection

**Figure 1:**
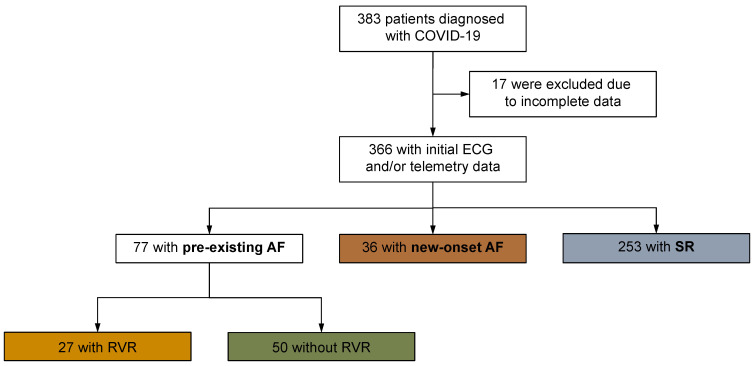
Flow chart of the study population. AF = atrial fibrillation, SR = sinus rhythm, RVR = rapid ventricular rate. This study included 383 patients hospitalized with COVID-19 infection; 17 patients had to be excluded from the statistical analysis due to insufficient data collection. The patient record was analyzed concerning AF, recording the documented electrocardiograms (ECG) and events in telemetric monitoring during the hospital stay. Patients without a history of AF and a documented sinus rhythm (*n* = 253) were assigned to the control group. Patients with AF (*n* = 117) were differentiated according to whether they had a history of AF (*n* = 77) or whether they had new-onset AF during hospitalization (*n* = 36). The former population was also considered regarding the question of whether a patient with previously known AF suffered RVR (*n* = 27) or had no such rhythm events during hospitalization (*n* = 50).

This study is a retrospective single-center study that included *n* = 383 patients hospitalized with COVID-19 infection at the Marburg University Hospital from 18 March 2020 to 10 April 2021 (Figure 1). Inclusion criteria were defined as the presence of PCR-confirmed SARS-CoV-2 infection and patient age >18 years. Data from the inpatient stay were analyzed. A small number of patients (*n* = 17) had to be excluded from the statistical analysis due to insufficient data collection. This left a collective of *n* = 366 patients to be examined, who were, on average, 70 years old and 62% male. The patient record was organized concerning AF, with documented electrocardiograms (ECG) and telemetric monitoring events recorded during hospitalization. For the following statistical surveys, the patient collective was divided into four groups. Patients without a history of AF and a documented sinus rhythm (*n* = 253) were assigned to the control group. Patients with AF (*n* = 117) were differentiated according to whether they had a history of AF (*n* = 77) or whether they had new-onset AF during hospitalization (*n* = 36). The focus of the statistical analysis is to compare the patients with new-onset AF (new-AF) with the group without AF and documented sinus rhythm (control group). To be as accurate as possible, cases of patients with existing paroxysmal or persistent AF but without documented episodes of rapid ventricular rate (RVR) defined as a heart rate > 100 bpm during COVID-19 infection (pre-AF without RVR, *n* = 50) and recurrent paroxysmal or persistent AF with RVR during COVID-19 (pre-AF with RVR, *n* = 27) were excluded from and compared with the control group. For all patients, traditional pre-existing conditions and cardiovascular risk factors, among others, were recorded and listed in Table 1. 

### 2.2. Ethical Statement

The study protocol and all experimental protocols were approved by the Ethics Committee of the Medical University of Marburg.

### 2.3. Laboratory Analyses

Routine blood chemistry parameters, including differential blood count, highly sensitive c-reactive protein (hs-CRP), interleukin 6 (IL-6), and procalcitonin (PCT) levels, were analyzed using fresh blood samples according to established methods at the local laboratories. 

### 2.4. Assessment of Potential Covariates

In this study, the demographic and clinical characteristics of the patients were recorded. Among other parameters, age, body weight (including body mass index (BMI)), smoking, previous diseases, and medication use were considered. BMI is calculated by dividing body weight in kilograms (kg) by the square of the patient’s height in meters (m^2^). Nicotine abuse was defined as smoking ten or more cigarettes per day. In case a patient had a blood pressure ≥140 mmHg systolic or ≥90 mmHg diastolic, or if there was antihypertensive treatment in the medical history, hypertension was defined in these patients. When assessing type II diabetes mellitus, the following parameters were evaluated: an HbA1c level ≥6.5% and hypoglycemic therapy. A total cholesterol level ≥240 mg/dL or a fasting triglyceride level ≥150 mg/dL and lipid-lowering drug treatment resulted in classification into the group of patients with hyperlipidemia if any of these parameters were present.

### 2.5. Endpoints and Collection of Data

The primary endpoint of the study is to assess intrahospital mortality in relation to the occurrence of AF in hospitalized patients with COVID-19 infection.

### 2.6. Statistical Analysis

Data were analyzed using IBM SPSS Statistics for Windows, version 26 (IBM corp., Armonk, NY, USA). *p*-values below 0.05 are interpreted as significant. Pairwise comparisons for mortality rate, intensive care unit (ICU) requirement, and ventilator requirement were calculated with Chi^2^ N-1 tests and corrected for multiple testing according to Bonferroni. Pairwise comparisons for the age of patients in each group were calculated using one-factor ANOVA and Games-Howell post hoc tests for heterogeneous variances. The comparisons for body mass index and length of stay were calculated with one-factorial ANOVA. If the ANOVA was significant, a Bonferroni correction was applied to the pairwise comparisons. The comparisons of the number of comorbidities and those of the blood values were calculated with t-tests (Satterthwaite correction for heterogeneous variances). Possible influences of cardinally scaled variables (in this case, the number of comorbidities and the observation of blood values) on a binary dependent variable (AF yes/no) were modeled as logistic regression. Concerning the table with the baseline characteristics, several statistical methods were used. Chi² tests (for 2 × 2 tables: Chi^2^ N-1 tests) were used for the correlations between categorical variables. If the sample size was small, the Fisher’s Exact Test was used. Mean comparisons between two groups were analyzed with t-tests; in the case of significant violations of the test requirements of the t-test, they were analyzed as Mann–Whitney tests.

## 3. Results

In this study, a total of *n* = 366 hospitalized patients with COVID-19 infection at a German university hospital were investigated.

**Figure 2:**
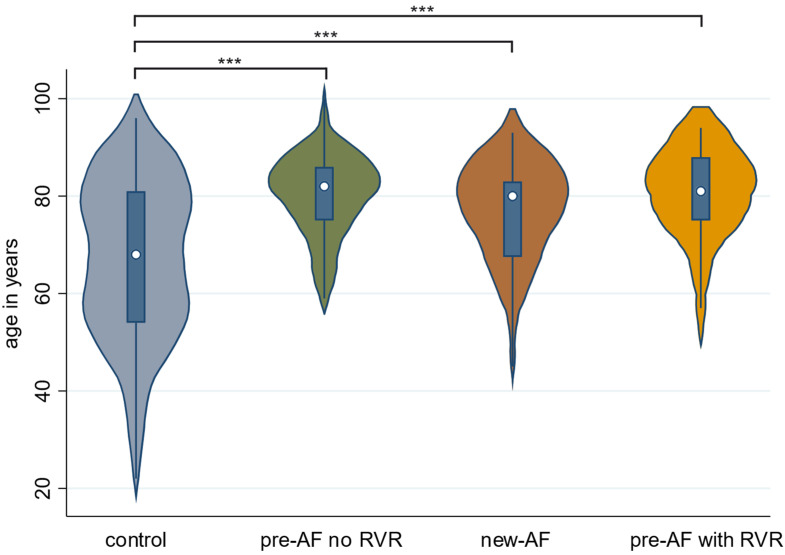
Age distribution of the individual study groups. *** *p* < 0.001. Patients with new-AF were, on average, 75.5 years old (SD ± 12.0); patients with preexisting AF and no RVR were 80.2 years old (SD ± 8.7); and patients with preexisting AF and RVR were 79.7 years old (SD ± 10.5). All patients in the groups with AF were significantly older than patients in the control group, who were, on average, 66.4 years (SD ± 17.0) old.

Patients with new-AF were, on average, 75.5 years old (SD ± 12.0), significantly older than patients in the control group, who were, on average, 66.4 years (SD ± 17.0) old (*p* < 0.001) (Figure 2). The patients with new-AF were 80.6% male. This is a significantly higher proportion than in the control group, where 61.7% of patients were male (*p* = 0.027). New-AF patients were hospitalized for a median of 11.5 days (IQR 4.0–21.0), while control group patients were hospitalized for 9 days (IQR 4.0–16.0 days). No significant differences were found when considering the length of stay and BMI in the different groups. Additional patient characteristics, particularly a breakdown of comorbidities, are shown in Table 1.

**Figure 3:**
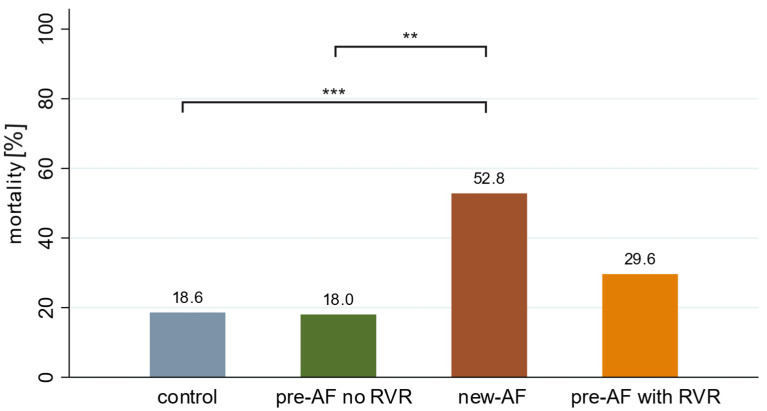
The intrahospital mortality rate of the individual study groups. ** *p* < 0.01, *** *p* < 0.001. In patients with new-AF, a mortality rate of 52.8% was found, which was significantly higher than the control (18.6%) and pre-AF no RVR (18%).

**Figure 4:**
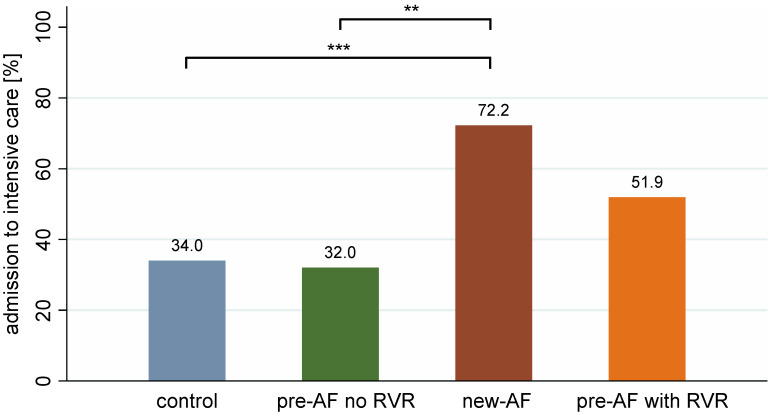
Percentage representation of the proportion of patients requiring intensive care. ** *p* < 0.01, *** *p* < 0.001. The significantly higher need for ICU treatment of 72.2% in patients with new-AF compared to 34% in the control group (*p* < 0.001) indicates a more severe disease course of COVID-19 pneumonia in AF.

In our study population, 36 patients (9.8%) had documented new-onset AF on admission ECG or during inpatient therapy. Furthermore, pre-existing paroxysmal/persistent AF was documented in the patient records of 77 patients (pre-AF). However, within this collective, relevant rhythm events with RVR during hospital stay were found in only 27 patients (pre-AF with RVR), and no relevant tachycardic phases during the hospital stay could be documented in 50 patients (pre-AF without RVR). It should be emphasized that, when analyzing mortality in the setting of COVID-19 in the different groups, new-onset AF is a predictor of mortality in hospitalized patients. In this regard, our studies showed that more than one in two of these patients did not survive hospitalization (mortality rate of 52.8%). The mortality rate is thus significantly higher compared to the other groups and in the control group (*p* < 0.001). In (Figure 3), for example, it amounts to only 18.6%. Interestingly, the pre-AF without RVR group shows comparable mortality to the control group (18.0%), and the pre-AF with RVR patients shows increased mortality compared to the control group (29.6%). A survival curve of the individual study groups is shown in the Supplementary Materials (Figure S1). The significantly higher need for the ICU treatment of 72.2% of patients with new-AF compared to 34.0% in the control group (*p* < 0.001) also indicates a more severe disease course of COVID-19 pneumonia in AF (Figure 4). To assess the severity of the COVID-19 disease in this study, it was assessed whether patients had oxygen requirements during hospitalization and/or required non-invasive or invasive ventilation. It was shown that there was a higher rate of oxygen demand and ventilatory therapy in new-AF and pre-AF with RVR. Here, 33.3% in the new-AF and 29.6% in the pre-AF with RVR versus 17.0% in the control group required invasive ventilation. However, the corresponding results did not show statistical significance in the analyses.

During data collection, comorbidities and cardiovascular risk factors were recorded, and baseline characteristics are summarized in Table 1. It was found that patients with new-AF were significantly more likely to have pre-existing renal insufficiency (38.9% vs. 15.8%, *p* < 0.001) and arterial hypertension (77.8% vs. 60.5%, *p* = 0.045) compared to the control group. Furthermore, in this study, creatinine levels were collected regularly at different time points during the observation interval. A highly significant association between elevated creatinine levels and new-onset AF was demonstrated in the group comparison between new-AF and control (*p* = 0.002).

Within the analyses, patients with new-AF and patients with pre-existing AF with RVR were found to have similar risk profiles and comparable outcome parameters (Table 1). Similarly, patients in the control group with documented sinus rhythm and patients with pre-existing AF without RVR were shown to differ little in terms of risk profile and outcome. Accordingly, patients with new-AF (*n* = 36) and pre-AF with RVR (*n* = 27) were combined as a common group in the analysis of comorbidities and blood parameters (*n* = 63) and compared against the remaining patients (*n* = 303). Patients with new-AF/pre-AF with RVR were found to have more comorbidities than the other patients (2.32 ± 1.33 vs. 1.88 ± 1.53, *p* < 0.05). However, an association between the number of comorbidities and AF is not statistically detectable [OR = 1.071 (CI 0.886–1.294); *p* = 0.480]. Examination of patients’ laboratory values on the admission day shows that in patients who have new-AF/pre-AF with RVR compared to the rest of the patients, the parameters CRP [OR = 1.005 (CI 1.002–1.008); *p* = 0.002] and PCT [OR = 1.295 (CI 1.048–1.599); *p* = 0.017] are significantly increased. Similar analyses of interleukin-6 levels and absolute monocyte and lymphocyte counts showed no statistically significant associations. Furthermore, the differential blood count analysis showed no significant differences in the studied collectives for the percentage and absolute populations of monocytes and lymphocytes.

## 4. Discussion

In this single-center study, we demonstrated that new-onset AF is an important prognostic factor in the setting of COVID-19. This correlation was already confirmed in larger meta-analyses [24]. It was shown that a relevant proportion of patients develop AF for the first time and that patients with pre-existing AF are comparable in outcome to this group depending on the occurrence of tachyarrhythmia phases with heart rates >100 bpm. Particular risk factors for the occurrence of AF were found to be advanced age, male sex, the presence of arterial hypertension, and renal insufficiency. These risk factors have already been found in the context of sepsis [25], and in our study, it was shown that in the group with new-onset atrial fibrillation, there is a predominance of the male sex (80.6%) and this group has the highest overall mortality. It is already known that men develop atrial fibrillation more frequently and at a younger age than women [26].

In the general population, the prevalence of AF is 0.95% and increases with age [27]. However, viral infections, such as COVID-19 [28], are known to significantly increase incidences. In addition, the interaction of the risk factors arterial hypertension and chronic kidney disease with new-onset AF is also well known [29,30]. The association with kidney disease may be related to the effect on the heart and blood vessels, as well as other factors such as electrolyte imbalances and inflammation. Several studies have shown that inflammatory responses in the body, such as sepsis, increase the risk of AF, and new-onset AF was associated with increased mortality [25]. An approximately fourfold increased risk of AF has been reported in the setting of pneumonia [31]. In addition, with special reference to COVID-19 pneumonia, a significantly increased risk for the occurrence of new-onset AF was found [32]. COVID-19, as a systemic inflammatory disease, causes an unexpectedly high prevalence of hyper coagulopathy and thrombosis with further thromboembolic events possible (e.g., mesenteric ischemia, myocardial infarction, ischemic stroke) [33]. In addition, the SARS-CoV-2 virus can directly damage the heart muscle, increasing the risk of the occurrence of AF [34]. In this context, ACE2 plays a central role in the pathogenesis of the virus. ACE2 is considered a necessary receptor for the invasion of human cells by SARS-CoV-2 and is found in many organs, including the heart, lungs, kidneys, and intestines [7].

Previous studies have shown, comparable with our results, that AF has been the most common cardiac arrhythmia associated with myocardial inflammation during the COVID-19 pandemic [35]. Although electrical and structural remodeling plays a key role in AF pathophysiology, the clinical presentation of COVID-19-associated AF is diverse, and the putative mechanisms and potential clinical implications remain unclear, but several possible factors have been proposed. COVID-19 is known to cause an excessive immune response, which can lead to systemic inflammation with high cytokine release. Some researchers have suggested that arrhythmias are not a direct result of SARS-CoV-2 infection, but inflammatory cytokines may mediate the development of arrhythmias in COVID-19 patients [28]. Other explanations for the association between COVID-19 and AF include disturbances in the autonomic nervous system, which controls heart rate and rhythm, and electrical and structural changes in the heart are suspected [36]. Viral myocarditis may also cause damage to the myocardium and increase the incidence of AF. COVID-19 is also associated with hypercoagulability, which can lead to blood clots in the heart and lungs. These clots can cause atrial fibrillation or exacerbate existing atrial fibrillation [37].

The focus of our results is on the significantly increased mortality in COVID-19 patients who developed new-onset AF during hospitalization. It should be emphasized that the data collection period of our study particularly included patients who had been infected with the alpha, beta, and delta variants of the COVID-19 virus [38]. Furthermore, it has already been described in the literature that pre-existing AF in COVID-19 patients is associated with increased mortality, but this did not differentiate more precisely concerning relevant tachycardic phases during hospitalization [39]. Here, our study provides new insights regarding the differentiation between patients with RVR and without RVR. We were able to extend the fact of increased mortality in patients with pre-existing AF to the fact that even in patients with a previously faded history of such arrhythmia events, new-onset AF significantly worsens the course and outcome of the disease. Although the values obtained did not show significant differences between the populations, the higher values for mortality and need for intensive care in patients with new-onset AF compared with patients with pre-existing AF are striking. A possible explanation for the higher number of patients with new-onset AF compared with other studies [39,40] is that our study population included only patients who were hospitalized for more than 24 h. In addition, the data were collected at a maximum-care hospital (university hospital); patients are older and more likely to have a critical status. Moreover, a patient population with more previous illnesses and a complicated course of the disease is to be expected. The first vaccinations against COVID-19 were only available in Germany from 26 December 2020, and initially in a very limited number. As a result, no patients with complete vaccination protection were included in the study during the observation period of this study until the beginning of April 2021, and therefore no statement can be made regarding the possible effects of COVID-19 vaccines.

Thus, it remains to be noted that the pathomechanism of AF in COVID-19 and pneumonia should be of great interest, as the outcome for patients is significantly worse. Overall, the presence of AF in COVID-19 patients is an important consideration for healthcare providers, and close monitoring and management of AF and its associated complications may be necessary to optimize outcomes. In addition, further studies should investigate whether an approach to prevent the interaction between pneumonia and AF can be found, as this could be a starting point for better treatment of patients with increased mortality. 

## 5. Strengths and Limitations

Due to the retrospective data analysis and the observational nature of the study, hidden confounders are possible. Furthermore, although the COVID-19 patients received ECG documentation on admission to the hospital, they routinely had continuous ECG monitoring only during their inpatient stay in the ICU or intermediate care unit. Accordingly, episodes of AF may not have been documented in some patients. It should also be noted that this study included only patients who were hospitalized with COVID-19 at a maximum-care hospital. Thus, the generalizability of our results to asymptomatic or symptomatic COVID-19 patients who were not hospitalized may be limited. The exact severity of SARS-CoV-2 infection depends on many factors and can vary widely due to preexisting conditions. Regarding the occurrence of atrial fibrillation, this fact must be considered a potential confounder. Furthermore, this is a single-center study and refers to a population in a specific geographic region. Initially, our statistical analysis did not distinguish between women and men with respect to mortality and the need for intensive care. Overall, no significant differences were demonstrated when women were considered individually in the comparison of the group between new-AF and control. However, it should be noted that the number of women was smaller, and further studies should examine whether gender-dependent differences exist.

So far, the COVID-19 pandemic has been characterized by recurrently emerging variants, some of which differ significantly in their transmissibility and disease severity. Accordingly, the results obtained here can only be applied to the currently predominant Omicron variant to a limited extent, and new data collection is required in this regard. In addition, there is now a very high level of infectious disease and vaccination in the population, and these differ significantly in relation to the survey period of this study.

## 6. Conclusions

This retrospective study found that in patients hospitalized for COVID-19, AF is associated with increased intrahospital mortality in men. In addition, men with new-onset AF, in particular, have a more severe course of the disease. In male patients with pre-existing AF, it was correlated with the presence of an increased heart rate >100 bpm. 

## Figures and Tables

**Table 1:** Baseline characteristics of study participants.

	Control Group	Pre-AF no RVR	*p*-Value	New-AF	*p*-Value	Pre-AF with RVR	*p*-Value
Number of COVID-19 patients ^1^	*n* = 253 (69.1%)	*n* = 50 (13.7%)		*n* = 36 (9.8%)		*n* = 27 (7.4%)	
Age ^2^	66.4 (17.0)	80.2 (8.7)	<0.001 ^+^	75.5 (12.0)	<0.001 ^+^	79.7 (10.5)	<0.001 ^+^
Male sex ^1^	156 (61.7%)	30 (60.0%)	0.826 *	29 (80.6%)	0.027 *	11 (40.7%)	0.036 *
Duration of stay ^3^	9.0 (4.0–16.0)	9.0 (4.0–20.0)	0.561 ^#^	11.50 (4.0–21.0)	0.188 ^#^	11.0 (3.0–19.0)	0.682 ^#^
Body mass index ^3^	27.8 (24.5–31.8)	28.4 (24.0–32.5)	0.852 ^#^	27.5 (24.4–32.2)	0.733 ^#^	27.8 (25.2–31.5)	0.861 ^#^
Diabetes mellitus type I ^1^	3 (1.2%)	0 (0%)	1 ^§^	1 (2.8%)	0.414 ^§^	0 (0%)	1 ^§^
Diabetes mellitus type II ^1^	66 (26.1%)	24 (48,0%)	0.002 *	14 (38.9%)	0.109 *	12 (44.4%)	0.043 *
Bronchial asthma ^1^	18 (7.1%)	3 (6.0%)	1 ^§^	0 (0.0%)	0.142 ^§^	1 (3.7%)	1 ^§^
COPD ^1^	18 (7.1%)	12 (24.0%)	0.001 ^§^	1 (2.8%)	0.485 ^§^	4 (14.8%)	0.247 ^§^
DVT/PE ^1^	21 (8.3%)	3 (6.0%)	0.777 ^§^	3 (8.3%)	1 ^§^	3 (11.1%)	0.714 ^§^
Kidney failure ^1^	40 (15.8%)	18 (36.0%)	0.001 *	14 (38.9%)	0.001 *	7 (25.9%)	0.182 ^§^
Arterial hypertension ^1^	153 (60.5%)	40 (80.0%)	0.009 *	28 (77.8%)	0.045 *	23 (85.2%)	0.012 *
Coronary heart disease ^1^	41 (16.2%)	21 (42.0%)	<0.001 *	5 (13.9%)	0.723 *	8 (29.6%)	0.106 ^§^
Hypercholesterolemia ^1^	32 (12.6%)	8 (16.0%)	0.523 *	5 (13.9%)	0.792 ^§^	5 (18.5%)	0.374 ^§^
Cardiomyopathy ^1^	28 (11.1%)	22 (44.0%)	<0.001 *	6 (16.7%)	0.403 ^§^	6 (22.2%)	0.115 ^§^
Nicotine abuse ^1^	36 (14.2%)	10 (20.4%)	0.271 *	1 (2.8%)	0.061 ^§^	3 (11.1%)	1 ^§^

^1^: *n* (%); ^2^: Mean (SD); ^3^: Median (IQR); ^+^: *t*-test (with Satterthwaite correction for heterogeneity of variances); ^#^: Mann–Whitney-Test; *: Chi^2^ N-1-Test; ^§^: Fisher’s exact test; Abbreviations: COPD: chronic obstructive pulmonary disease; DVT: deep vein thrombosis; PE: pulmonary embolism.

## Data Availability

The data presented in this study are available upon request from the corresponding author. The data are not publicly available for ethical reasons.

## Supplementary Materials

The following supporting information can be downloaded at: https://www.mdpi.com/article/10.3390/jcm12103500/s1. Figure S1: Survival curve of the individual study groups (pre-AF with RVR, new-AF, pre-AF no RVR, control). AF = atrial fibrillation, RVR = rapid ventricular rate.
