# Supplementary material for: New-Onset Atrial Fibrillation in the Setting of COVID-19 Infection Is a Predictor of Mortality in Hospitalized Patients: CovAF-Study
Source: J Clin Med. 2023 May 16;12(10):3500. doi: 10.3390/jcm12103500 (PMC10218921; doi:10.3390/jcm12103500)
Supplement: Supplementary file 1 [file jcm-12-03500-s001.zip › jcm-2371988-supplementary.pdf]

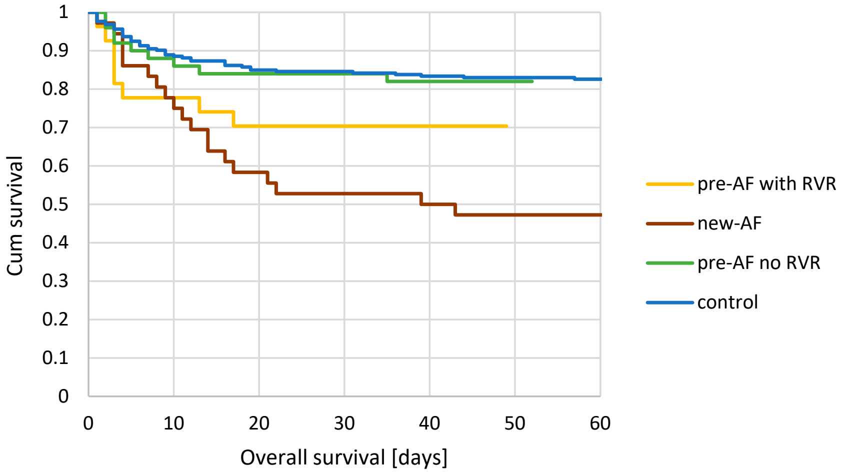

Supplementary Figure S1. Survival curve of the individual study groups (pre-AF with RVR, new-AF, pre-AF no RVR, control). AF = atrial fibrillation, RVR = rapid ventricular rate.
